# Transgluteal access for computed tomography-guided percutaneous puncture of prostatic abscesses

**DOI:** 10.1590/0100-3984.2019.0050

**Published:** 2020

**Authors:** Rômulo Florêncio Tristão Santos, Reinaldo Santos Morais Neto, Fábio Galvão Vidal, Luiz Augusto Morelli Said, Thiago Franchi Nunes

**Affiliations:** 1 Universidade Federal de Mato Grosso do Sul (UFMS), Campo Grande, MS, Brazil.; 2 Hospital Regional de Mato Grosso do Sul (HRMS), Campo Grande, MS, Brazil.

## INTRODUCTION

Prostatic abscess is a rare clinical condition that can result in serious complications such as urosepsis and death if it is not properly diagnosed and treated^(1)^. Once the diagnosis is established, the standard treatment options are the administration of antibiotics, open perineal drainage, and transurethral resection of the prostatic abscess. Currently, however, minimally invasive procedures such as needle aspiration guided by imaging methods are well established in interventional radiology practice and are preferable to conventional methods, with low rates of complications and satisfactory therapeutic results^(1-8)^.

Percutaneous drainage of pelvic abscesses are challenging because of the interposition of a large number of anatomical structures. Consequently, various routes of access and drainage techniques have been described^(2,9)^. To ensure that the procedure is successful, it is vital to plan the access route, which requires detailed knowledge of the pelvic anatomy. There are five major routes to approach deep pelvic lesions^(9)^: transabdominal (anterior and lateral), anterolateral extraperitoneal, transvaginal, transrectal, and transgluteal.

In transgluteal access, the patient is usually placed either in the prone position or in the lateral position. This technique is also known as transischial access, because the needle will pass through the greater sciatic foramen. When possible, the needle will transfix the sacrospinal ligament, located below the level of the piriform muscle, in order to avoid injuring blood vessels of the gluteal region and sacral plexus that are located anteriorly to the muscle. Lesions eligible for the employment of this technique are those posterior to the urinary bladder and adnexal masses^(9,10)^. This access has the advantage of avoiding piercing of the peritoneum, minimizing the risk of injury to bowel, bladder, and iliac vessels, and giving the needle stable access through a static muscle mass, free from the respiratory movements of the abdominal wall. The disadvantage lies in the use of the uncomfortable prone position, which complicates respiration and management of the anesthesia.

## PROCEDURE

Reviewing imaging examinations before the procedure is of paramount importance for its technical success. To localize the prostatic abscess and plan a safe path for the puncture needle, the procedure should be based on the best anatomical definition.

**Figure 1:**
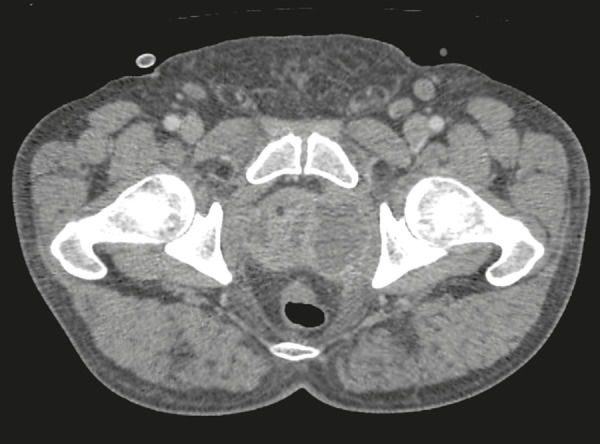
Axial contrast-enhanced computed tomography of the pelvis, showing a prostatic abscess.

**Figure 2:**
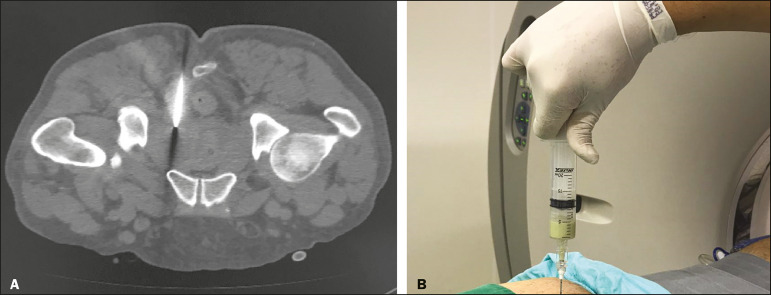
Computed tomography-guided transgluteal access performed with the patient in the prone position (**A**) and aspiration of purulent content (**B**).

**Figure 3:**
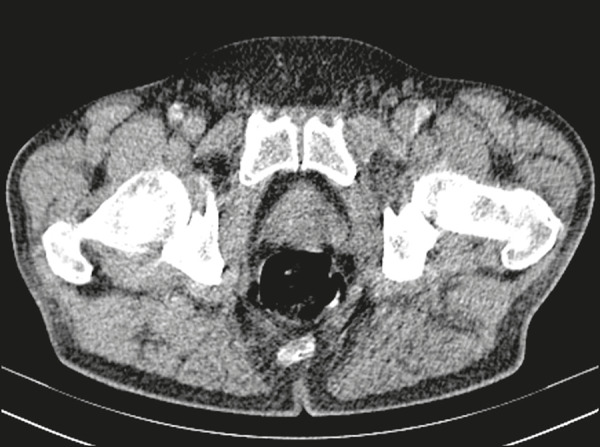
Computed tomography performed four weeks after the treatment, with no residual or new prostatic collections.

Computed tomography-guided transgluteal access performed with the patient in the prone position (Figure 1), under local anesthesia, allows the surgeon to view the 17-gauge aspiration needle from its insertion into the skin through to the pelvic cavity (Figure 2). The needle will pass through the greater sciatic foramen and transfix the sacrospinal ligament, located below the level of the piriform muscle, to avoid injuring blood vessels of the gluteal region and sacral plexus that are located anteriorly to the muscle^(9)^. Although it is not a widely used technique, aspiration of the abscess contents, followed by flushing with 50 mL of 0.9% saline solution (using a Luer Lock 10 mL syringe) and antibiotic therapy, has a high success rate (Figure 3) and few complications when compared with transurethral resection of the abscess and perineal insertion of a large-caliber catheter for long-term drainage to control the residual infection^(2,9-11)^. The advantages of the former procedure include presenting fewer potential complications and being performed under local anesthesia and sedation, which minimizes the risks of adversities after general anesthesia, especially in severely ill patients, as well as the possibility of repeating the procedure in the event of relapse. Ideally, it should be performed by interventional radiologists with training in percutaneous procedures^(9-11)^.
